# Zero dispersion Kerr solitons in optical microresonators

**DOI:** 10.1038/s41467-022-31916-x

**Published:** 2022-08-13

**Authors:** Miles H. Anderson, Wenle Weng, Grigory Lihachev, Alexey Tikan, Junqiu Liu, Tobias J. Kippenberg

**Affiliations:** 1 Institute of Physics, Swiss Federal Institute of Technology Lausanne (EPFL), 1015 Lausanne, Switzerland; 2 Present Address: Institute for Photonics and Advanced Sensing (IPAS), and School of Physical Sciences, The University of Adelaide, Adelaide, SA 5005 Australia

**Keywords:** Nonlinear optics, Solitons, Frequency combs

## Abstract

Solitons are shape preserving waveforms that are ubiquitous across nonlinear dynamical systems from BEC to hydrodynamics, and fall into two separate classes: bright solitons existing in anomalous group velocity dispersion, and switching waves forming ‘dark solitons’ in normal dispersion. Bright solitons in particular have been relevant to chip-scale microresonator frequency combs, used in applications across communications, metrology, and spectroscopy. Both have been studied, yet the existence of a structure between this dichotomy has only been theoretically predicted. We report the observation of dissipative structures embodying a hybrid between switching waves and dissipative solitons, existing in the regime of vanishing group velocity dispersion where third-order dispersion is dominant, hence termed as ‘zero-dispersion solitons’. They are observed to arise from the interlocking of two modulated switching waves, forming a stable solitary structure consisting of a quantized number of peaks. The switching waves form directly via synchronous pulse-driving of a Si_3_N_4_ microresonator. The resulting comb spectrum spans 136 THz or 97% of an octave, further enhanced by higher-order dispersive wave formation. This dissipative structure expands the domain of Kerr cavity physics to the regime near to zero-dispersion and could present a superior alternative to conventional solitons for broadband comb generation.

## Abstract (web summary)

Here, the authors find the missing link for soliton microcombs that exist at the boundary where the group velocity dispersion of light changes sign: zero-dispersion solitons. The resulting microresonator frequency comb, based in Si3N4, spans almost an octave.

## Introduction

Currently, the field of research in optically driven Kerr nonlinear resonators and dissipative structure formation has been largely focused on the paradigm of the bright dissipative soliton^1–3^. Bright dissipative solitons (DS) can be thought of as a particular variety of localized dissipative structure, solitary pulses that retain their shape due to the counter-balance between anomalous dispersion and nonlinearity, and who have a fixed amplitude determined by the driven-dissipative parameters of the Kerr cavity environment^4^. Bright DS have been widely studied experimentally in multiple material platforms^1^, and have been demonstrated as a desirable candidate for numerous integrated frequency comb-based applications such as massively parallel telecommunications^5^ and LiDAR^6^, astro-spectrometer calibration^7,8^, dual-comb spectroscopy^9^, and also for metrology enabled by self-referencing such as absolute frequency synthesis^10^ and towards optical atomic clocks^11^. Across optical physics, DS have been observed in nonlinear systems such as mode-locked lasers, and transverse laser cavities^4,12,13^, and more widely as basic structures in nonlinear dynamical systems as diverse as plasma physics, neuron propagation, and chemical reaction systems^14–16^.

In opposition to bright DS have been dark dissipative structures, commonly called “dark pulses”, which conversely exist in Kerr cavities possessing normal dispersion^17–21^. These dark pulses (alternatively termed as “platicons”^22^) are in fact formed by the interlocking of two separate switching waves (SW), connecting the high and low stable states of the bistable Kerr cavity^23^. Compared to bright dissipative solitons, they have been found to possess an intrinsically higher optical conversion efficiency between the input pump and the generated light as normal dispersion allows more comb lines far from the pump to be on resonance^24^. As such, they have been proposed as a superior alternative to bright DS for applications, which require the generation of strong comb lines near to the pump center, and have been demonstrated as a source for massively parallel telecommunications^25^. Switching waves have been classed more generally as “domain walls” connecting two stable homogeneous states in driven-dissipative systems, seen in *χ*^(2)^ optical parametric oscillators^26,27^, semiconductor lasers^28^, birefringent optical fibers^29,30^, and more widely in hydrodynamic systems and more^31^.

In this work, we report the experimental observation of zero-dispersion dissipative solitons (ZDS), which exist at the crossing point between conventional dissipative solitons and switching waves, excited in the region of vanishing second-order dispersion (SOD) giving way to pure- or dominant- third-order dispersion (TOD)^32^. The ZDS appears as a self-stable, multi-peaked pulse structure. In prior theoretical and numerical studies, such structures we identify here as ZDS have been termed as bright solitons existing at zero SOD^33,34^ (for instance as a soliton “doublet”^35^) and, in more recent work more explicitly described as strongly modulated, bright interlocked switching waves^36–38^. Across all of these prior studies, the terms “platicon”, “bright soliton”, and “locked switching waves” have all been used together interchangeably to describe precisely the same entity. In this work, we seek to draw a concise and practical distinction from the single-peaked plain soliton on the one hand, and the free-moving switching waves on the other, in order to make an unambiguous entity that exists in the regime near zero dispersion that has a multi-peaked structure. Hence, the specific term “zero-dispersion soliton” is presented here.

**Fig. 1:**
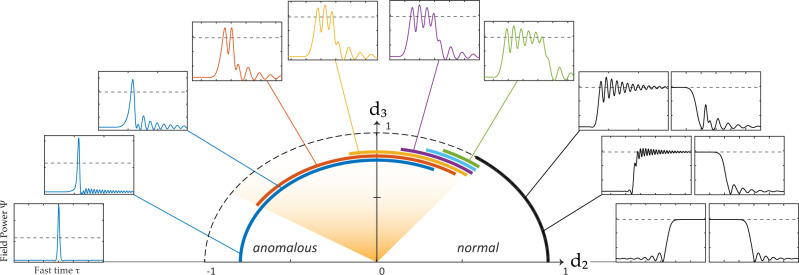
Localized dissipative structures in the second-/third-order dispersion (${{{{{{d}}}_{2}/d}}}_{{{{{{{{\bf{3}}}}}}}}}$) plane. Clockwise from left: conventional dissipative solitons, dissipative solitons with dispersive-wave tails, zero-dispersion solitons with quantized periods (orange area), switching waves with dispersive-wave tails, conventional switching wave. Dashed gray line in outer figures represent the CW high-state solution. Thick bands represent the existence range of structures in the circular path. Further analysis and extended version of this figure with a video available in Supplementary Info.

As depicted in Fig. 1, ZDS exist as a family of solutions, for generalized localized dissipative structures, as one traces a circular path of dispersion in the SOD/TOD plane. This ZDS family (here obtained through numerical simulations), is shown to occupy the connection between the diametrically opposed cases, of conventional dissipative solitons on the anomalous dispersion regime, and switching waves on the normal dispersion regime. As one approaches the zero-dispersion region in the center, in both cases of DS and SW, the dispersive-wave tail (otherwise known as Cherenkov radiation)^39–41^ becomes increasingly dominant until it becomes an essential part of the structure^33^, and stable quantized multi-peaked pulses become accessible. We define the two boundaries of this region of ZDS to be where solitons may become multi-peaked on the anomalous side, and where the two switching wave fronts become locked together on the normal side.

**Fig. 2:**
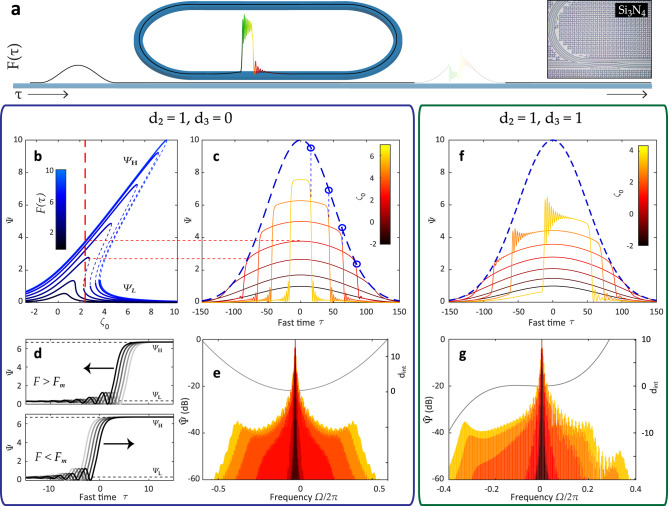
Excitation of switching waves inside pulse-drive envelope. **a** Principle of pulse-driven Kerr cavity dissipative structure formation, with image of resonator with coupling section. **b**–**e** SW formation in pure normal dispersion. **b** Contour of the bistable intracavity CW solutions, plotted for increasing local value of the pump *F*(*τ*), with stable (unstable) solution in solid (dashed) line. Red-dashed line marks detuning after wave-breaking occurs. **c** Development of intracavity field (red-yellow) within the pulse envelope (blue dashed) with increasing detuning *ζ*_0_. Red-dotted lines connect one field slice with the CW solution distribution in **b**. Maxwell points on the pulse envelope marked with circles. **d** Expanding (top) and contracting (bottom) high-state (dashed) under CW driving. **e** Spectra of the fields from **c**, and integrated dispersion operator. **f**, **g** SW formation with strong third-order dispersion. **f** Intracavity field within the pulse envelope with increasing detuning *ζ*_0_. **g** Spectra of the fields from **f**.

Experimentally, it has remained an open question how these ZDS could be generated. In this work, we target the formation of ZDS on the normal SOD side of this plane. We find that this can be accomplished through first generating switching waves efficiently via wave-breaking using synchronous pulse-driving of the Kerr cavity^42^ (Fig. 2(a)), initially demonstrated in fiber cavities^43,44^, and proposed for microresonators^37^, and observing how these SWs coalesce into single ZDS states at high pump-cavity detuning.

## Results

### Theory

To analyze the optical structures introduced in this work in a simple and universal fashion, we first consider an optical system described by the dimensionless Lugiato-Lefever Equation (LLE)^45,46^, now with a non-CW driving term *f*(*τ*):

1 $$\frac{\partial \psi }{\partial t^{\prime} }=\, \left(-{d}_{1}\frac{\partial }{\partial \tau }-i{d}_{2}\frac{{\partial }^{2}}{\partial {\tau }^{2}}+{d}_{3}\frac{{\partial }^{3}}{\partial {\tau }^{3}}\right)\psi \\ \,+(i|\psi {|}^{2}-i{\zeta }_{0}-1)\psi+\sqrt{{F}_{0}}f(\tau)$$

Here, the form taken by the field solutions are determined solely by the driving strength *F*_0_ and detuning *ζ*_0_, as well as three parameters *d*_*l*_ describing the relative contributions of the first three orders of dispersion^33^. For simplicity, we set the SOD parameter *d*_2_ = 1 throughout this work, which corresponds to normal dispersion. Thus, *d*_3_ describes the contribution of TOD relative to *d*_2_, and the first-order dispersion *d*_1_ corresponds to the offset in group-velocity between the cavity field *ψ*(*τ*) and the static frame of the pulse-driving term *f*(*τ*). These key parameters can be converted into real experimental values for the laser and the resonator by using the transformations given in the Methods section.

Firstly, it is necessary to investigate direct SW formation by pulse-driving, in the simplified case of pure SOD (*d*_1_ = *d*_3_ = 0). We choose a value of *F*_0_ = 10, equivalent to several Watts peak power in our Si_3_N_4_ devices, a typical operating point for practical dissipative structure formation in experiment, and we set a Gaussian pulse as the driving function $f(\tau )=\exp (-{\tau }^{2}/{\tau }_{p}^{2})$, with pulse duration *τ*_*p*_ = 100 so as to ensure any SW is significantly shorter in duration than the envelope of the driving pulse (which is true also in our experiment), and so the driving parameter at each SW location can be considered approximately CW. The detuning is swept linearly from some value *ζ*_0_ < 0 up to *ζ*_0_ = 10 (5 × the resonator linewidth). Owing to the Kerr nonlinearity, the cavity resonance becomes tilted as a result of the additional phase acquired over propagation for higher pump power. This creates an expanding range of bistability for steady-state CW solutions, the high-state *ψ*_*H*_ and low-state *ψ*_*L*_ (the intermediate state is inaccessible). These solutions can be found from Eq. (2),(3) (see Methods). In Fig. 2(b), these bistable solutions are plotted for the different power levels that exist across the envelope of the driving pulse.

In Fig. 2(c) (with spectra in 2(e)) we show the intracavity field solutions Ψ = ∣*ψ*∣^2^ at different values of *ζ*_0_ found using the split-step method^47^ (see Methods). For this direction in *ζ*_0_, the field initially follows the high-state solution *ψ*_*H*_(*τ*) of the bistable resonance. As *ζ*_0_ crosses 0, there begin to exist parts of the intracavity field where the local Kerr resonance-shift at the edges of the pulse-drive *F*(*τ*) is insufficient to sustain the high-state *ψ*_*H*_(*τ*) (an example detuning of which is marked by the red-dashed line in Fig. 2(b)). Here, the field outside this point falls to the low-state *ψ*_*L*_(*τ*) while the field further inside the pulse background stays on *ψ*_*H*_(*τ*) creating the SW that connects the two states^26^.

From here, the two SW locations *τ*_SW_ follow a location within the pulse-drive envelope *F*(*τ*) = *F*_*m*_, which previous theoretical works on SW stability have termed as the “Maxwell Point”^23,28^, until at *ζ*_0_ ≈ 7 where there exists no *F*(*τ*) > *F*_*m*_ causing the SWs to meet each other and annihilate, failing to reach their theoretical maximum detuning at *ζ*_0_ = *F*_0_ = 10. The stability of the SW fronts within the pulse envelope after formation is due to the effective “outward pressure” manifesting on *ψ*_*H*_. SWs possess an innate group-velocity offset depending on the value of *F* and *ζ*_0_^43^, where the *ψ*_*H*_ tends to undergo expansion, with the SWs moving outward, when the driving term is larger than a certain value *F*(*τ*) > *F*_*m*_ for a fixed detuning *ζ*_0_ (see Fig. 2(d)). When *F*(*τ*) < *F*_*m*_, the *ψ*_*H*_ contracts and the SWs move inward. Accepting this, it becomes clear that if any high-state *ψ*_*H*_ existed within a pulse-drive envelope, whose peak *F*_0_ > *F*_*m*_, it would undergo expansion until its SW fronts reached a point where *F*(*τ*_SW_) = *F*_*m*_ and stop.

Considering now a Kerr cavity possessing strong TOD, we choose *d*_3_ = 1. Fig. 2(f, g) presents an analogous scenario to Fig. 2(b–e), now with TOD enabled. We see in Fig. 2(f) that the leading-edge SW front (left-hand side) has acquired an upper-state oscillation that corresponds to an enhancement of the spectrum (Fig. 2(g)) on the negative frequency side. This enhancement is due to the return to zero of the integrated dispersion function *d*_int_ = *d*_2_Ω^2^ + *d*_3_Ω^3^ (Ω being the dimensionless angular frequency)^41^. This asymmetry in the spectral profile imparts a positive group-velocity shift to the leading-edge SW front, and causes the entire upper-state to collapse at a lower detuning than in the case of pure SOD.

**Fig. 3:**
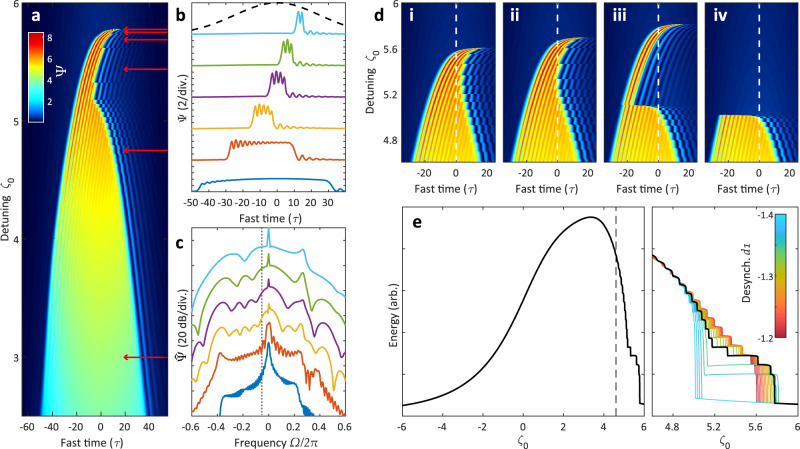
Simulation: zero-dispersion soliton formation via desynchronized pulse-driving. *F*_0_ = 10, *d*_2_ = 1, *d*_3_ = 1 **a** Intracavity field as detuning *ζ*_0_ is increased. *d*_1_ = − 1.34 **b** Individual time domain and **c** frequency domain of the fields in **a** (red arrows). Pulse-drive envelope *F*(*τ*) marked by dashed line, and zero-dispersion frequency marked by dotted line, with normal dispersion on the righthand side. **d** Alternative field formation with (i) *d*_1_ = − 1.20 (ii) *d*_1_ = − 1.29 (iii) *d*_1_ = − 1.36 (iv) *d*_1_ = − 1.37. White-dashed line marks the pulse-drive center. **e** (left) Total intracavity energy from **a** (right) Zoom of the “step” feature, plotted for different values of desynchronization *d*_1_. The black line corresponds to the field given in **a**.

Overall, the flatness of the dispersion profile on negative frequencies has heavily skewed the generated spectrum to the one side resulting in a negative shift to the group velocity for the SW structure as a whole inside *F*(*τ*). Naturally, introducing a counter-acting group-velocity shift in the form of a negative *d*_1_ term should help contain the structure within the center of *F*(*τ*) as the detuning increases. This scenario is presented in Fig. 3. By now setting *d*_1_ = − 1.34, the time-frame of the cavity field continually moves forward in fast time *τ* (here to the left), keeping both SWs near to the center of the pulse envelope *F*(*τ*) preventing early collapse. The SW fronts meet together now at *ζ*_0_ = 5.1, where a significant event occurs. Instead of eliminating each other as in the case of pure SOD, the SWs become locked to each other based on the bonding of the down-SW to the modulated wave of the up-SW forming the stable ZDS.

This structure, forming based on interlocking switching waves, is similar but different to the formation of previously researched “dark pulses”^20^. Such dark pulses form from the locking of the modulations of the low-state *ψ*_*L*_ and can form in the case of pure SOD, whereas these bright ZDS necessarily require strong TOD so that a sufficiently powerful modulation exists on the high-state *ψ*_*H*_. These ZDS further distinguish themselves from the SW state, which exists here at lower detunings for *ζ*_0_ < 5.1, in that the SW is bound overall by the left and right Maxwell points of the pulse-drive envelope *F*(*τ*), whereas the ZDS is self-stable akin to a DS, and can freely exist across the broad background of the pulse-drive. As with pulse-driven conventional DS, the ZDS moves itself towards a single trapping position on one edge of *F*(*τ*)^42,48,49^. In this example, the trapping position is on the left edge.

We term the complete structure as a ZDS^(*n*)^, with *n* individual peaks. As the detuning here increases past *ζ*_0_ > 5.1, the structure (plotted specifically on levels 3–6 of Fig. 3(b)) undergoes progressive collapses reducing its multi-peaked periodicity initially from *n* = 5, down to 2. In the frequency domain (correspondingly in Fig. 3(c)), we can determine the value *n* by the number of spectral periods between the pump and what was initially the SW dispersive wave, here on the left end of the spectrum. It must be pointed out that the bulk of the soliton component of the spectrum continues to exist in the anomalous dispersion region on the left, due to the “recoil” induced by the new powerful dispersive-wave component on the right^33,39^. This is one important explanation as to how a soliton can still exist, allowed by the counter-acting balance of dispersion and nonlinearity, while still being pumped from the normal dispersion region.

Figure 3(d) shows how varying the group-velocity shift *d*_1_ (or desynchronization in terms of pulse-driving) gives rise to a varying maximum detuning for ZDS^(*n*)^ existence, and with different preferred *n*. Here (with particular attention to Fig. 3(d-iii)), the ZDS^(3)^ follows its trapping position on the left-hand slope until it crosses the center line where a trapping position no longer exists and decays, following an asymmetrical trajectory reminiscent of recent studies on conventional dissipative solitons^48^. The cavity energy trace, plotted in Fig. 3(e) for all values of *d*_1_ in the vicinity, shows the asymmetrical unfolding of the characteristic “step” feature we should expect to see in experiment.

### Experimental results

**Fig. 4:**
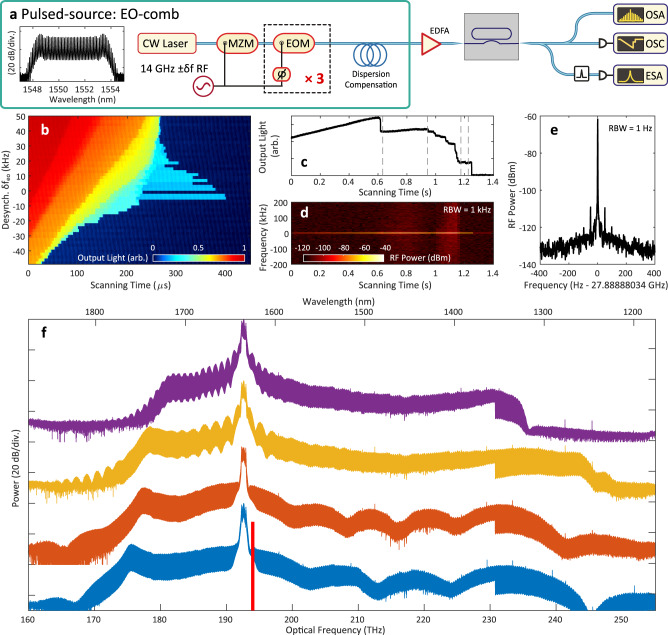
Experimental pulse-driven switching wave and zero-dispersion soliton formation, MR1. **a** Setup, featuring the EO-comb as a pulsed-source. MZM: Mach-Zehnder modulator, EOM: electro-optic modulator, EDFA: erbium-doped fiber amplifier, ESA: electronic spectrum analyzer, OSA: optical spectrum analyzer, OSC: oscilloscope. The input pulse train is coupled into and out of the microresonator chip via lensed fibers. Left-inset: Spectrum of the 14 GHz EO-comb before amplification. **b** Spectrogram of the step feature for different desynchronization about 27.888880 GHz. **c** Microresonator transmission (with DC value subtracted), with detunings from (f) marked with dashed lines. **d** Spectrogram of the repetition-rate beatnote during the laser scan in **c**. **e** Long-term beatnote measurement of the final comb state. **f** Stages of comb/spectrum formation in descending order of detuning (40 dB vertical offsets). Red block marks spectral filter for beatnote measurement.

The pulse-drive source (as shown in Fig. 4(a)) is provided in the form of an electro-optic comb (EO-comb)^42,50^, providing pulses with a minimum duration of 1 ps (see Methods for details), and whose repetition rate is finely controlled by an RF-synthesized signal *f*_eo_. The cavity platform of choice for the experiment is the chip-based Si_3_N_4_ microresonator, in this case having a native FSR of 27.88 GHz. The EO-comb repetition rate is set to exactly half this at *f*_eo_ = 13.944 GHz due to RF transmission limitations. Other than a factor-2 reduction on conversion efficiency due to only half the lines being coupled to the cavity, the experiment behaves the same as one that is fully synchronous and we can disregard the excess comb lines. Two microresonators (referred to as MR1 and MR2) in particular are used to generate ZDS, having two slightly different dispersion profiles causing the formation of ZDS^(*n*)^ of different *n*. Their measured dispersion parameters are given in result figures further below.

Starting with MR1, in order to ensure that a full range of formation behavior is observed, and spectral extent maximized, the average pulse-power coupled to the resonator is set to *P*_av_ = 350 mW (12 pJ pulse energy at 28 GHz), approximately 20 times higher than the observed minimum comb generation threshold. The experiment proceeds in a similar way as in the theory section, typical for localized dissipative structure generation in Kerr cavities and particularly in pulse-driven soliton generation^42,49,51^. The exact native FSR of the microresonator is first found by varying the input repetition-rate *f*_eo_ until the expected unfolding of the ZDS “step” is observed (Fig. 4(b)). Here, we see an asymmetrical extension of the step vs. the relative desynchronization *δf*_eo_ = *f*_eo_ − *D*_1_/2*π*, as expected based on Fig. 3(e), although with slightly different precise form due to unaccounted for higher-order effects (the absence of a step at *δf*_eo_ = 0 is a coincidence based on shot-to-shot statistical variation of formation probability). Based on this measurement, we find an optimum *f*_eo_ = 27.88888 GHz, with a locking range for ZDS on the order of ± 10 kHz.

For this value of *f*_eo_, the EO-comb seed laser frequency *ω*_*p*_ is tuned slowly across a resonance frequency *ω*_0_ from the blue-detuned side to the red-detuned side (such that *δω* = *ω*_0_ − *ω*_*p*_ > 0 by convention^2^) towards the region of Kerr bistability. Fig. 4(c) plots the output light from the microresonator during this scan, and at the same time the RF repetition-rate beatnote of the ZDS is recorded (Fig. 4(d)). From this we are able to see that throughout this scan over resonance, the RF repetition-rate beatnote shows low noise. We pause the scan of the laser at four example locations here in order to observe the steady-state solution of the SW and ZDS states. Here the comb spectra measured at the OSA are plotted in Fig. 4(f) at four detunings in descending order, after the SW is formed. Qualitatively, the results behave as the simulations in Fig. 3(c) predict. Firstly, over the first two rows, we see the spectrum grow wider and with a sharper dispersive-wave (DW1) located from 182 to 179 THz. Importantly, we see the spectral interference fringes either side of the pump (spaced by ≈ 1 THz on the first spectrum) increase their period as detuning is increased, indicating the two SW fronts are moving together within the pulse-drive envelope. In the last two rows, we see the SWs have coalesced into the ZDS^(5)^, a 5-period structure, then reducing to a ZDS^(4)^, each time moving the location of DW1 further to low frequencies. Stationary states existing in between the 2nd and 3rd rows were not able to be accessed due to them being thermally unstable. In 100% of experimental generations of ZDS in this way, only a single ZDS structure was ever formed. This is naturally as a result of the fact that just two SW fronts are generated at the wave-breaking stage with pulse-driving, which go on to lock together forming a single ZDS.

The long-term beatnote measured at this final state, plotted in Fig. 4(e), is highly stable, inheriting the low-offset phase noise of the *f*_eo_ as supplied by the RF synthesizer. This confirms that the ZDS has temporally locked to the driving pulse over the long-term, just as a conventional bright dissipative soliton would^42,49^. The ZDS state was able to exist in the microresonator for tens of minutes, eventually collapsing due to uncontrolled thermal drift of the cavity resonance mode away from the laser center frequency.

**Fig. 5:**
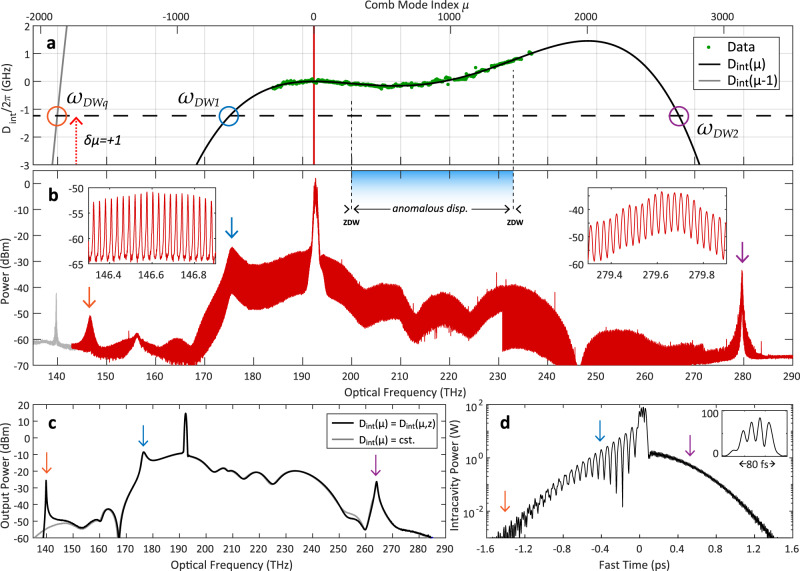
Octave spanning zero-dispersion soliton spectrum, MR1. **a** Experimental measurement of the resonator integrated dispersion *D*_int_, along with the spectrally extended fitted solution, and solution shifted by + *D*_1_. Pump frequency (and pump detuning) marked by the vertical red line (horizontal dashed line). Phase-matched locations of dispersive waves marked by circles, with momentum mismatch in dotted red arrow. **b** Measured spectrum of ZDS^(4)^, with dispersive waves marked with arrows corresponding to the circles in **a**. Insets show individual comb lines. The left-most trace is in gray to indicate it is the second-order diffraction spectrum of DW2, and not genuine. **c** Frequency domain and **d** time domain simulation of ZDS^(4)^ with dispersive-wave tails marked. The close agreement of the central spectral fringes between the experiment and simulation gives us confidence that we are observing a four-peak, 80 fs pulse (showed in linear scale inset).

Figure 5 analyses this final structure in greater detail. We characterize the broadband dispersion profile of the MR1 using a cascaded three-laser swept spectroscopy technique^52^. In Fig. 5(a) we plot the measured integrated dispersion profile *D*_int_ = *ω*_*μ*_ − *ω*_0_ − *μD*_1_, representing the frequency deviation of each resonator mode *μ* from the uniform FSR grid spaced by *D*_1_ (where the pump mode corresponds to *μ* = 0). This data is fitted to a fourth-order polynomial centered at *ω*_0_ = 2*π* × 192.3 THz where *D*_int_ ≈ *μ*^2^*D*_2_/2 + *μ*^3^*D*_3_/6 + *μ*^4^*D*_4_/24 (*D*_2_/2*π* = − 3.17 kHz, *D*_3_/2*π* = 13.8 Hz, *D*_4_/2*π* = − 15.9 mHz, all ± 5%). In dimensionless parameters (see Methods), for a fixed *d*_2_ = 1 we have a value *d*_3_ = 0.38. The pump frequency detuning − *δω*/2*π* = − 1.2 GHz, which we obtain from live cavity phase-response measurements^53^, is also marked. In Fig. 5(b) the entire spectrum of the ZDS^(4)^ is plotted and features several dispersive waves (DW), the spectral locations of which can be predicted based on where *D*_int_(*μ*) = − *δω*. The predicted DW locations do not match perfectly with experiment however, but this can be explained by the bandwidth-limited dispersion measurement with unknown higher-order values for *D*_5_, *D*_6_ and so forth.

The first, DW1 at 176 THz, will always occur for ZDS formation due to the requirement for powerful TOD. The second, DW2 at 280 THz, has occurred due to the overall normal fourth-order dispersion of the waveguide^54^, but is not required for ZDS formation. The additional dispersive wave, termed DWq, occurs where the optical comb modes have wrapped by *D*_1_ so that *D*_int_(*μ*) + *D*_1_ = *D*_int_(*μ* − 1) = − *δω* or that, by shifting by one FSR, the linear wave at DWq has accrued a 2*π* phase shift relative to the pump wave. This phase-wrapping is commonly known to allow the formation of so called “Kelly” sidebands in soliton fiber lasers^55^. Owing to the longitudinal momentum mismatch *δμ* = + 1 between the coupled linear wave and the ZDS comb lines, quasi-phase matching is required to bridge this gap^56^. This particular microresonator features a brief “mode-stripping” section where the waveguide width rapidly tapers down to a narrow width in order to stop any higher-order spatial modes from propagating^57^, where the waveguide dispersion changes sharply. This intra-roundtrip disturbance provides phase modulation to the linear wave at DWq, and is more than sufficient to enable quasi-phase matching to stimulate resonant radiation. This effect has been observed in microresonators with a similar intra-roundtrip modulation of the waveguide width^58^, and has long been observed in fiber-based Kerr resonators with longitudinally varying dispersion^44,59^. Figure 5(c, d) shows numerical LLE simulations using the real experimental parameters of MR1 (see Methods), demonstrating close agreement with the form taken by the spectrum corresponding to a ZDS^(4)^ as shown in Fig. 5(d). In Fig. 5(c), both simulation results taking into account either a constant or an oscillating intra-roundtrip dispersion *D*_int_(*z*), with DWq appearing only in the latter case. Further simulations and analysis of DWq is presented in the supplementary info.

**Fig. 6:**
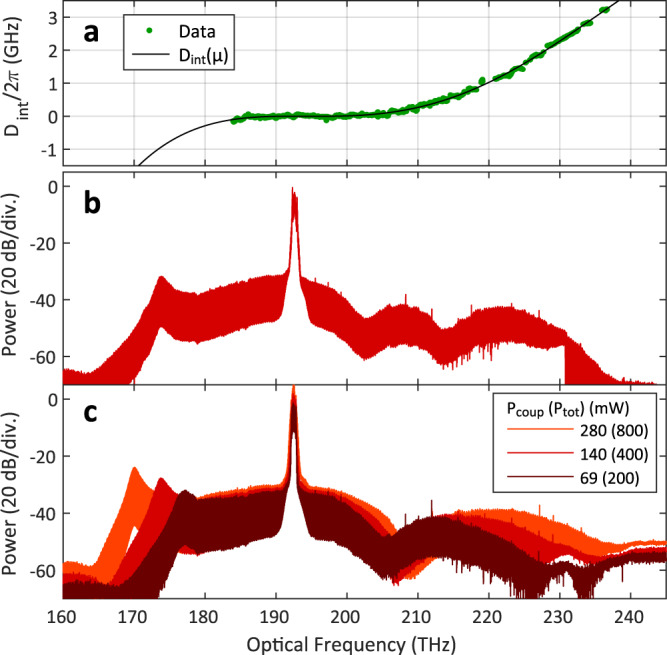
Experimental zero-dispersion soliton formation, MR2. **a** Experimental measurement of the resonator integrated dispersion *D*_int_, and spectrally extended fitted solution. **b** ZDS^(3)^ comb. **c** ZDS^(2)^ combs, measured at maximum detuning using three pump powers, noted as effective average power coupled to ring (total power towards chip).

In order to observe ZDS^(*n*)^ of lower *n* we move to MR2, which has its zero-dispersion wavelength closer to the pump wavelength at 1560 nm. Here, the dispersion parameters (Fig. 6(a)) are fitted to be *D*_2_/2*π* = − 848 Hz, *D*_3_/2*π* = 12.8 Hz, *D*_4_/2*π* = − 15.9 mHz, ± 5%, corresponding to dimensionless parameters *d*_2_ = 1 and *d*_3_ = 2.11, further into the zero-dispersion regime (Fig. 1). According to Fig. 1 and predictions given by Parra-Rivas et al.^36^, the existence range for ZDS^(*n*)^ of lower *n* is greater in this regime, and so are more likely to form within typical driving parameters. In this microresonator, with the same generation method as above in MR1, we generate ZDS^(3)^ and ZDS^(2)^ in Fig. 6(a) and (b), respectively. In this microresonator, we do not observe the same DW2 or DWq as in microresonator 1. For ZDS^(2)^, we present spectral measurements taken using three increasing input pump powers, each enabling an increased maximum detuning *δω*. As shown, as available power is increased, the overall spectral profile expands, with both DW1 on the left and the anti-dispersive wave on the right moving outwards, in a similar manor as for conventional dissipative solitons^60^.

## Discussion

In this work, we have experimentally synthesized a class of localized dissipative structures, that we term the zero-dispersion soliton. In terms of figures of merit, the generated ZDS^(2−5)^-based combs presented here are extremely substantial in terms of the product of their total bandwidth and their total line-count which, as far as we are aware, is a record for a single-structure in a microresonator. The central body of the ZDS^(4)^ comb in MR1 spans over 76 THz (1830 and 1260 nm), accounting for more than 2700 comb teeth, spaced by a detectable 28 GHz repetition rate. When including the DW features, the final bandwidth becomes 136 THz or 97% of an octave. As the repetition rate is directly detectable on photodiode, a future work with fine-tuning of the microresonator dispersion may enable *f*-2*f* self-referencing with a single microcomb^10^. We have further demonstrated the direct generation of switching waves via pulse-driving, creating a highly smooth ultra-broadband microcomb under normal dispersion conditions. Such normal dispersion-based microcombs have thus far only been formed in Si_3_N_4_ via modulation instability enabled by spatial mode-coupling^20^, necessitating an extra coupled-microresonator ring with integrated heaters in order to be deterministic^61^.

The formation of ZDS-based microcombs more generally has expanded the domain of microcomb generation towards the region of both normal dispersion and zero-dispersion, previously not often considered ideal. This lifting of strict requirements for anomalous dispersion may give greater flexibility in the Si_3_N_4_ fabrication process going forward. The result also demonstrates not only that microcomb generation can be achieved in a straight-forward fashion in such waveguide resonators with normal-to-zero dispersion, but that it may be most preferable for highly broadband comb generation due to the superior flatness of the comb in the SW regime, as well as the lack of a high-noise chaotic phase and multi-soliton formation as compared to its anomalous dispersion-based counterpart.

In terms of physics, the zero-dispersion soliton can be seen as a bright pulse-like structure, which constitutes a link between SW-based and soliton-based localized dissipative structure. Such a structure, the zero-dispersion soliton, defines itself in the regime where third-order dispersion becomes a dominant influence relative to second-order or conventional group-velocity dispersion, and where the dispersive-wave components become an essential part of the structure rather than a perturbation. The fact that the ZDS may exist in either anomalous or normal dispersion^38^ (referring to Fig. 1) presents an ambiguity in explanation for the structure’s existence, between being two interlocked switching waves in normal dispersion, or being a multi-peaked dissipative soliton in anomalous dispersion. Further investigation and analysis of ZDS existence will be needed to gain full understanding on this. More widely, this experimental observation may trigger further fundamental research on the nature of dissipative Kerr solitons and switching waves under one umbrella.

Note: we would like to acknowledge a parallel work by Li et al. completed during preparation for this manuscript, which observed the same ZDS structure in fiber-based Kerr cavities, including the single-peak soliton in both anomalous and normal second-order dispersion^62^.

## Methods

### Theory and simulation

The homogeneous solutions to the intracavity field Ψ_CW_ = ∣*ψ*_CW_∣^2^, graphed in Fig. 2(b) for different local pump strength *F*(*τ*) = *F*_0_*f*^2^(*τ*), is first obtained from the real roots of the cubic polynomial derived from Eq. (1) at equilibrium and with all dispersion *d*_*l*_ = 0^46^, and subsequently the complex field solution from the resonance condition,

2 $${{{\Psi }}}^{3}-2{\zeta }_{0}{{{\Psi }}}^{2}+({\zeta }_{0}^{2}+1){{\Psi }}-F(\tau )=0$$

3 $${\psi }_{{{{{{{{\rm{CW}}}}}}}}}=\frac{i\sqrt{F(\tau )}}{{{{\Psi }}}_{{{{{{{{\rm{CW}}}}}}}}}-{\zeta }_{0}+i}$$

with Ψ_*H*_ and Ψ_*L*_ being the top and bottom solution, respectively. The simulation presented in Fig. 2 was calculated via the split-step method with a change in detuning rate $d{\zeta }_{0}/dt^{\prime}=0.01$, far slower than the cavity photon lifetime, to ensure SWs reached equilibrium at each stage. In Fig. 3, $d{\zeta }_{0}/dt^{\prime}=0.000625$ to allow the ZDS to remain at their trapping/equilibrium position during he detuning increase. The pulse-drive width *τ*_*p*_ = 50.

### Experiment

The EO-comb is comprised of a CW laser, followed by an intensity modulator and three phase modulators, driven by an RF signal generator (Rhode & Schwarz SMB100A), generating 50 spectral lines spaced by *f*_eo_ = 13.944 GHz. The waveform is compressed in time through linear dispersion made from 300 m of standard SMF-28 and 5 m of dispersion-compensating fiber, yielding pulses of minimum duration 1 ps as confirmed by frequency-resolved optical gating (FROG). The Si_3_N_4_ microresonators MR1 and MR2 used in this experiment have been fabricated with the photonic Damascene process^63^ with a 2350 × 770 nm^2^ cross-section, and possess a peak probable cavity linewidth of *κ*/2*π* = (*κ*_0_ + *κ*_ex_)/2*π* = 208 MHz and 150 MHz (with external coupling rate *κ*_ex_/2*π* = 155 MHz and 120 MHz) for MR1 and MR2, respectively. Their measured dispersion *D*_int_ is expanded in the main text. Effective power coupled to resonator as quoted above exclude chip insertion loss of 1.6 dB and half of the 14 GHz comb lines not coupled to the resonator modes at 28 GHz. The RF beatnote measurement in Fig. 4(d, e) derives from approximately 11 filtered comb lines outside of the EO-comb spectrum.

### Full system model

The experimental microresonator results are described by the full LLE with real parameters

4 $$\frac{\partial A(t,T)}{\partial t}=	{{{{{{{\mathcal{F}}}}}}}}\left[i\left(\delta \omega+\mu \cdot 2\pi \delta \,{f}_{{{{{{{{\rm{eo}}}}}}}}}+{D}_{{{{{{{{\rm{int}}}}}}}}}(z,\,\mu )\right){\tilde{A}}_{\mu }\right]\\ 	 -\frac{\kappa }{2}A+i{g}_{0}|A{|}^{2}A+\sqrt{\frac{{\kappa }_{{{{{{{{\rm{ex}}}}}}}}}{P}_{0}}{\hslash {\omega }_{0}}}{f}_{p}(T)$$

acting on photon field *A*(*t*, *T*) over slow/laboratory time *t* and fast time *T* in the co-moving frame of the intracavity field circulating at *D*_1_, with frequency domain counterpart ${\tilde{A}}_{\mu }$ at discrete comb line indices *μ*. Included with the linear phase operators *D*_int_ and *δω* is the input pulse desynchronization *δf*_eo_. The nonlinear coupling parameter *g*_0_/2*π* = 0.056 Hz (see supp. info for further on this).

For the simulation presented in Fig. 5(c, d), we set *δω*/2*π* = 900 MHz, and the input pulse profile ${f}_{p}(T)=\exp (-{T}^{2}/{T}_{p}^{2})$ with *T*_*p*_ = 0.85 ps, *P*_0_ = 1.8 W, and static desynchronization *δf*_eo_ = 150 kHz. In order to stimulate the quasi-phase-matched wave at DWq, we set ${D}_{{{{{{{{\rm{int}}}}}}}}}(z,\,\mu )={D}_{{{{{{{{\rm{int0}}}}}}}}}(\mu )(1+0.2\cos (\delta \mu \cdot 2\pi z/L))$, with *δμ* = 1 representing a single longitudinal-mode modulation in the dispersion operator for the resonator of length *L*. All of the real parameters are related to the dimensionless parameters by the following: $t^{\prime}=\frac{\kappa }{2}t$, $\tau={D}_{1}\sqrt{\frac{\kappa }{{D}_{2}}}T$, $\psi=\sqrt{\frac{2{g}_{0}}{\kappa }}A$, ${d}_{l}=\frac{2}{\kappa }\frac{{D}_{l}}{l!}{\left(\frac{\kappa }{{D}_{2}}\right)}^{\frac{l}{2}}$ for *l* = 1–4, ${\zeta }_{0}=\frac{2\delta \omega }{\kappa }$, ${F}_{0}=\frac{8{\kappa }_{{{{{{{{\rm{ex}}}}}}}}}{g}_{0}}{{\kappa }^{3}\hslash {\omega }_{0}}{P}_{0}$.

## Supplementary information

Supplementary Information

Description to Additional Supplementary Information

Video for Figure 1: Dispersion Rotation

## Data Availability

The data that support the plots within this paper are available at 10.5281/zenodo.6759788^64^. Any other data and findings of this study are available from the corresponding author upon reasonable request.

## Code availability

The simulation code used to produce the plots within this paper are available at 10.5281/zenodo.6759788^64^.
