# Supplementary material for: Zero dispersion Kerr solitons in optical microresonators
Source: Nat Commun. 2022 Aug 13;13:4764. doi: 10.1038/s41467-022-31916-x (PMC9376110; doi:10.1038/s41467-022-31916-x)
Supplement: Supplementary file 1 — Supplementary Information [file 41467_2022_31916_MOESM1_ESM.pdf]

Supplementary Information to Zero-dispersion Kerr solitons in optical microresonators

Miles H. Anderson,[1] Wenle Weng,[1,2] Grigory Lihachev,[1] Alexey Tikan,[1] Junqiu Liu,[1] and Tobias J. Kippenberg[1,*]

[1]*Institute of Physics, Swiss Federal Institute of Technology Lausanne (EPFL), Lausanne, CH-1015, Switzerland*
[2]*Present address: Institute for Photonics and Advanced Sensing (IPAS), and School of Physical Sciences, The University of Adelaide, Adelaide, 5005, South Australia, Australia.*

I. EXTENDED LOCALISED DISSIPATIVE STRUCTURE FIGURE AND SUPPLEMENTARY VIDEO

Attached with supplementary material is a video of a simulation depicting the results of Fig. 1 in the main text, and shown again here expanded in Fig. S.1. This simulation is calculated based on Eq. 1, the dimensionless Lugiato Lefever Equation (LLE),

$$\frac{\partial\psi}{\partial t'} = \left(-id_2\frac{\partial^2}{\partial\tau^2} + d_3\frac{\partial^3}{\partial\tau^3}\right)\psi + (i|\psi|^2 - i\zeta_0 - 1)\psi + \sqrt{F_0}f(\tau) \tag{1}$$

with second and third order dispersion parameters changing over the long time t, where $d_2(t) = \cos(2\pi t/t_p)$ and $d_3(t) = \sin(2\pi t/t_p)$, for one period of $t_p = 11,520$, covering the dispersion semi-circle shown in both directions, forwards and backwards. This time-scale is long enough that the solutions found can be considered to be the long-term stable solutions. The simulation is pulse-driven with $F_0 = 10$ at detuning $\zeta_0 = 5$, with pulse-drive envelope $f(\tau) = \exp(-\tau^2/\tau_p^2)$ with $\tau_p = 30$, sufficient to sustain a localised dissipative structure at all dispersion values. The simulation begins with a switching wave within the pulse envelope at pure normal dispersion ($d_2 = 1, d_3 = 0$), moves around the dispersion circle to ($d_2 = -1, d_3 = 0$), and then continues back to terminate at ($d_2 = 1, d_3 = 0$), revealing the hysteresis of the stable zero-dispersion solitons (ZDS) for the forwards and backwards direction. From this, we can see the start and end points for the ZDS$^{(n)}$, including $n = 1$ to $n = 6$. In the video, live plots include the instant dispersion value in the top-right polar-plot and the total intracavity energy value, showing the sharp transitions between states. Further included are live plots of the frequency domain (top-right), real and imaginary values for the time domain field (bottom-left), and the time domain power field (bottom-right). Note: the centre-of-energy of the pulse waveform is purposefully shifted in fast time dynamically to stay in the centre of the pulse-drive envelope, in order to maintain its existence.

For the results shown in the video, we can highlight specific solutions in Fig. S.1, marked in the top semi-circle diagram with points (A-J), along with their existence ranges within this dispersion circle for this value of F_0 and ζ_0. The solutions are plotted in the fast time and frequency domain, and the locus of the time domain field in the complex plane. Beginning with (A) we have the dissipative soliton solution found at pure-anomalous dispersion (Note: in the video, the live solution is in a breathing state, however, the initial stable solution is plotted here). Next at (B), we see the dissipative soliton with dispersive wave tail (Cherenkov radiation). In row (ii) we see the dispersive wave tail in how the field spirals towards the homogenous low-state solution ψ_L. At (C), the solution of the strongly perturbed dissipative soliton able to exist in the *normal dispersion* plane is found. This structure was not able to be obtained in this experiment, but was observed in a concurrent work on these zero-dispersion solitons in fibre-based cavities [1].

At (D), (E), (F), and (G), we find the multipeaked ZDS$^{(n)}$ states for n = 2, 3, 4, and 6 (omitting 5 for brevity). In rows (ii), the complex field is shown to follow a spiraling excursion around the high-state ψ_H, with each spiral forming each of the individual peaks in the time domain pulse. The resulting frequency domain on rows (iii) is highly-structured, but it appears that the number of spectral periods on the negative-frequency side (opposite to the soliton dispersive wave) corresponds to the number of peaks in the time domain pulse. At (H), the two ends of the ZDS in fast time un-lock from each other, resulting in two switching waves that move outward. What used to be the individual peaks of the ZDS has become the high-state dispersive wave tail, seen in row (ii) as a spiral towards the high-state ψ_H. At (I) and (J), this high-state dispersive wave disappears, leaving only the low-state dispersive waves on each switching wave.

A useful given explanation for the zero-dispersion solitons seen in this work [2, 3] is that they are two interlocked switching waves forming a self-stable bright pulse. However, an important observation of this simulation which may

* tobias.kippenberg@epfl.ch

complicate this explanation is the fact that, for this example value of F_0 and ζ_0, the 'single-roll' soliton solution is able to exist in the normal dispersion plane (as previously predicted in [4]), and the ZDS$^{(2)}$ and ZDS$^{(3)}$ solutions are likewise able to exist in the anomalous dispersion plane. This would seem to suggest that the single-roll soliton solution could also be thought of as a single-period interlocked switching wave, despite existing continuously with the conventional pure-anomalous soliton solution. On the other hand, the ZDS$^{(2)}$ for instance, existing continuously in anomalous dispersion, could be thought of as the dual-cycle form of the dissipative soliton, given that the single soliton is thought of as a single period of the patterned solution (modulation instability, or Turing rolls) that connects the low state ψ_L with the high state ψ_H in this regime [5]. It is within this *ambiguity* that we define the family for ZDS solutions to be unique from the conventional dissipative solitons and switching waves that exist in the pure or near-pure second-order dispersion regimes.

II. SUPPLEMENTARY MICRORESONATOR AND EXPERIMENTAL DETAILS

We are able to track the laser-cavity detuning by performing continuous probing of the linear cavity resonance with phase-modulation sidebands, as depicted in the experimental setup in Fig. S.2, with the response fed back into a vector-network analyser [6]. The '$\mathcal{C}-$resonance' (termed as such in[6]), shown in the plot in Fig. S.2, marks the detuning for the switching wave state (SW) and the ZDS$^{(4)}$ state found in main text Fig. 4 and 5.

The microresonator in use for the experiment is designed with a non-uniform waveguide width, creating the perturbation to the travelling ZDS enabling the quasi-phase matching dispersive wave highlighted in main text Fig. 5. The principle waveguide width is 2,350 nm, over a length of 5,140 µm, with two turning circles of radius 200 µm. However the waveguide tapers down to 450 nm wide and back over a length of 300 µm (see diagram in Fig. S.3), originally for the purpose of expelling higher-order spatial modes from the resonator to prevent high-quality inter-mode coupling [7]. Finite-element method calculations of the dispersion value β_2 for each waveguide width are given in Fig. S.3, including the aggregate dispersion for the 'mode-stripping' tapered section, as well as the average dispersion for the microresonator as a whole over the total roundtrip. A waveguide height of 710 nm gives results closest to that found in the experiment, hence we infer that waveguide height. As shown, the dispersion value changes hugely for a brief moment during propagation about the resonator, and we take this as justification for the varying dispersion introduced in the following section.

III. GENERALIZED MEAN-FIELD MODEL AND QUASI-PHASE MATCHING SIMULATION

The full experimental ZDS structure can be simulated with the full LLE for intracavity photon flux $A(t,T)$ over the co-rotating fast time co-ordinate T, changing over long time t:

$$\frac{\partial A(t,T)}{\partial t} = \mathcal{F}\Big[i\big(\delta\omega + \mu\cdot 2\pi\delta f_{\mathrm{eo}} + D_{\mathrm{int}}(z,\mu)\big)\tilde{A}_\mu(t)\Big] - \frac{\kappa}{2}A + ig_0|A|^2A + \sqrt{\frac{\kappa_{\mathrm{ex}}P_0}{\hbar\omega_0}}f_p(T) \tag{2}$$

with $A(t,T)$ related to the frequency domain over comb line index μ by

$$A(t,T) = \sum_\mu \tilde{A}_\mu(t)e^{-i\mu D_1 T} \tag{3}$$

The field A experiences loss determined by the cavity linewidth $\kappa = \kappa_{\mathrm{ex}} + \kappa_0$, and frequency-dependent detuning contained in the terms inside the Fourier transform $\mathcal{F}[\ \ ]$. These terms include the zeroth-order $\delta\omega = (\omega_0 - \omega_p)$ representing the detuning between the driving carrier frequency ω_p and the central resonator mode ω_0, the first-order correction to the cavity repetition rate δf_{eo} determined by the *mismatch* between the natural cavity repetition rate and the input EO-comb pulse repetition rate where $\delta f_{\mathrm{eo}} = f_{\mathrm{eo}} - D_1/2\pi$, and the remaining higher-order integrated dispersion operator D_{int}, to be described further below. The nonlinear coupling term $g_0 = \hbar\omega_0^2 v_g^2 n_2/(cA_{\mathrm{eff}}L)$, representing the Kerr frequency shift per photon, for a cavity of effective volume $A_{\mathrm{eff}}L$, material nonlinear refractive index n_2, and waveguide group velocity v_g. The cavity is driven with a pulse $f_p(T) = \exp(-T^2/T_p^2)$ with peak power P_0 through cavity coupling rate κ_{ex}.

We define the higher-order dispersion term up to the fourth-order, where

$$D_{\mathrm{int0}}(\mu) = \frac{D_2}{2}\mu^2 + \frac{D_3}{6}\mu^3 + \frac{D_4}{24}\mu^4 \tag{4}$$

and we let the magnitude of the dispersion operator vary over the non-rotating resonator spatial co-ordinate z, where

$$D_{\text{int}}(z,\mu) = (1 + \Delta(z))D_{\text{int0}}(\mu) \tag{5}$$

with z being equivalent to the *slow time* dimension t, in that $z \equiv tLD_1/2\pi \mod L$. Even though the variation of the dispersion operator over the roundtrip of the resonator could be described fully by the functions given in Fig. S.3, for simplicity and for demonstrative purpose, we shall take a case where $\Delta(z) = 0.2\cos(\delta\mu \cdot 2\pi z/L)$ with $\delta\mu = 1$, representing a small, single-longitudinal mode variation to the field $A(t,T)$. The average value of dispersion is unchanged so that $\overline{D_{\text{int}}(z)} = D_{\text{int0}}$. All parameter values are given in the table below.

The simulation presented in Fig. S.4 (expanding on that presented in main text Fig. 5(c,d)) is carried out with the split-step Fourier method [8, 9], at a step rate per roundtrip $(\Delta z/L)^{-1}$ of 16 for a duration of 800 roundtrips, sufficient to capture both the fast and slow change in the spectrum. In this way, we are able to 'observe' the ZDS with temporal super-resolution, at a faster rate than in experiment at multiple samples per roundtrip. The sub-roundtrip dynamics of the ZDS can reveal the nature of intracavity quasi-phase matching, in a similar way to what has been observed in previous simulations for Kerr cavity solitons in optical fibre-loop cavities using a 'lumped' propagation model [10, 11]. A ZDS$^{(5)}$ is formed on top of the $f_p(T)$ background by hard excitation, and the simulation presented begins after a time where the state has relaxed into an equilibrium position.

In Fig. S.4(a) we see the T vs. t image of the ZDS in propagation, over a truncated 8 roundtrips for clarity. Taking the Fourier transform on T gives us the spectrum behaviour over t in Fig. S.4(b), where we now see more clearly how two quasi-phase matched dispersive waves on the far left and far right have revealed themselves which appear to be oscillating at the period of the dispersion variation. Taking the Fourier transform across the long time dimension t brings us to Fig. S.4(c), the 'slow frequency' vs. 'fast frequency' image. Here, the stable frequency comb spectrum $\tilde{A}_\mu$ of the ZDS$^{(5)}$ is seen as a thin line horizontally across the centre. Background shot noise entered into the cavity field every simulation step is able to reveal the '$\mathcal{C}-$resonance' at every comb line index μ, which corresponds to the total dispersion spectrum operator (the Fourier transform term in Eq. 2). The two locations where this resonance spectrum intersects with $\tilde{A}_\mu$ give us our conventional zeroth-order phase-matched dispersive waves at $\mu = -550$ and $\mu = 2,600$.

At the bottom of the image, the dispersion spectrum reaches one free-spectral range (FSR) down at $-D_1/2\pi = -27.89$ GHz at two more locations, intersecting with the first-order momentum-mismatched spectrum $\tilde{A}_{\mu-1}$. As depicted in Fig. S.4(e) for the left-hand case, the dispersion spectrum at ω_μ at $\mu = -1,878$ finds itself phase-matched with the comb line 1 longitudinal mode back, at $\mu = -1,879$. Normally, without any periodic perturbation to the ZDS as it circulates the resonator, this comb line would be non-resonant due to this momentum mismatch, but thanks to the $\Delta(z)$ term with $\delta\mu = 1$, this mismatch is able to be bridged. The same simulation run with $\Delta = 0$ gives *no* resonant radiation at these locations. This energy transfer between adjacent modes is also revealed by the simulated comb repetition rate beatnote captured in Fig. S.4(d), calculated from the long term amplitude of the first-order sideband of the optical power spectrum as

$$\mathcal{F}_t\left[I_{\mu=1}(t)\right] \tag{6}$$

where

$$I_\mu(t) = \mathcal{F}_T\left[|A(t,T)|^2\right] \tag{7}$$

showing two small sidebands spaced by the FSR. A surf plot of the same frequency vs. frequency image is shown in Fig. S.4(f) from a raised perspective in order to better reveal the spectral amplitude of $\tilde{A}_\mu$ and $\tilde{A}_{\mu-1}$. In particular, it reveals how the $\tilde{A}_{\mu-1}$ spectrum is resonant at the quasi-phase matched locations. While this presented analysis shows we should expect to see both quasi-phase matched dispersive waves at $\mu = -1,878$ and $\mu = 3,700$, only the former was strong enough to be visible in the experiment.

Parameter	Value
n_2	2.4×10^{-19} m^2/W
L	5140 μm
A_{eff}	1.4 μm^2
$\omega_p/2\pi$	192.3 THz
$\delta\omega/2\pi$	900 MHz
$D_1/2\pi$	27.88888 GHz
δf_{eo}	-130 kHz
$D_2/2\pi$	-3.17 kHz
$D_3/2\pi$	13.8 Hz
$D_4/2\pi$	-15.9 mHz
$\kappa_0/2\pi$	60 MHz
$\kappa_{\mathrm{ex}}/2\pi$	155 MHz
P_0	1.8 W
T_p	0.85 ps
$\delta\mu$	1
Δ_0	0.2

[1] Z. Li, Z. Li, Y. Xu, Y. Xu, S. Coen, S. Coen, S. G. Murdoch, S. G. Murdoch, M. Erkintalo, and M. Erkintalo, Optica **7**, 1195 (2020).

[2] P. Parra-Rivas, D. Gomila, and L. Gelens, Physical Review A **95**, 053863 (2017).

[3] V. E. Lobanov, N. M. Kondratiev, A. E. Shitikov, R. R. Galiev, and I. A. Bilenko, Physical Review A **100**, 013807 (2019).

[4] C. Milián and D. V. Skryabin, Optics Express **22**, 3732 (2014).

[5] P. Parra-Rivas, D. Gomila, M. A. Matías, S. Coen, and L. Gelens, Physical Review A **89**, 043813 (2014).

[6] H. Guo, M. Karpov, E. Lucas, A. Kordts, M. H. P. Pfeiffer, V. Brasch, G. Lihachev, V. E. Lobanov, M. L. Gorodetsky, and T. J. Kippenberg, Nature Physics **13**, 94 (2017).

[7] A. Kordts, M. H. P. Pfeiffer, H. Guo, V. Brasch, and T. J. Kippenberg, Optics Letters **41**, 452 (2016).

[8] G. Agrawal", in *"Nonlinear Fiber Optics (Fifth Edition)"*, "Optics and Photonics", edited by G. Agrawal" ("Academic Press", "Boston", "2013") "fifth edition" ed., pp. "27 – 56".

[9] T. Hansson, D. Modotto, and S. Wabnitz, Optics Communications **312**, 134 (2014).

[10] J. K. Jang, M. Erkintalo, S. G. Murdoch, and S. Coen, Nature Photonics **7**, 657 (2013).

[11] M. Anderson, Y. Wang, F. Leo, S. Coen, M. Erkintalo, and S. G. Murdoch, Physical Review X **7**, 031031 (2017).

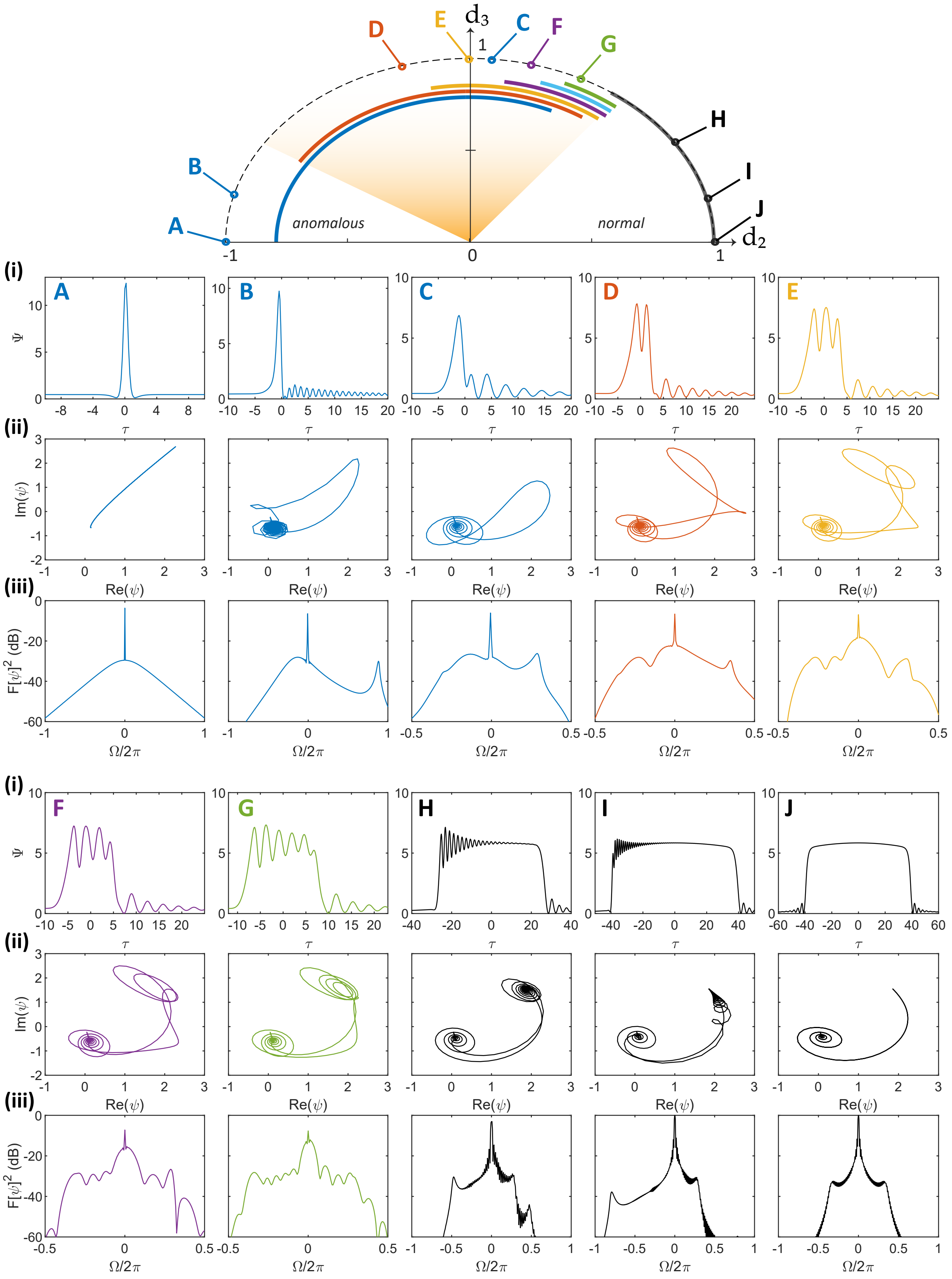

FIG. S.1. **Family of solutions for localized dissipative structures in the second-/third-order dispersion ($\mathbf{d_2/d_3}$) plane.** Top: Unit circle for d_3 vs. d_2 with solutions (A)-(J) marked. Thick bands indicate corresponding existence ranges, demonstrating 'snaking' solutions predicted in [2]. (A)-(C): Dissipative soliton solutions. (D)-(G): Zero-dispersion soliton solutions. (H)-(J): Switching wave solutions. Rows (i): Time domain field $\Psi(\tau) = |\psi(\tau)|^2$ vs. time τ. (ii) Complex field path in the time domain. (iii): Frequency domain.

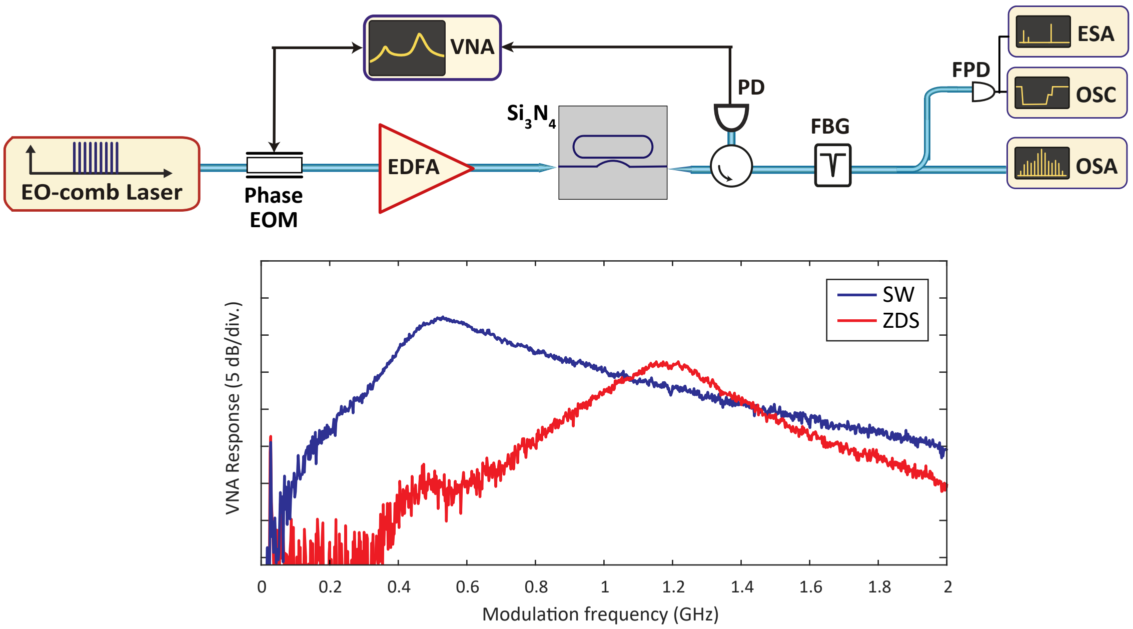

FIG. S.2. **Laser-cavity detuning measurement.** Top: experimental setup. EOM: electro-optic modulator, EDFA: erbium-doped amplifier, VNA: vector-network analyser, PD: photodiode, FBG: fibre-bragg grating, FPD: fast photodiode. Bottom: VNA-response during experiment, showing $\mathcal{C}-$resonance for different cavity states.

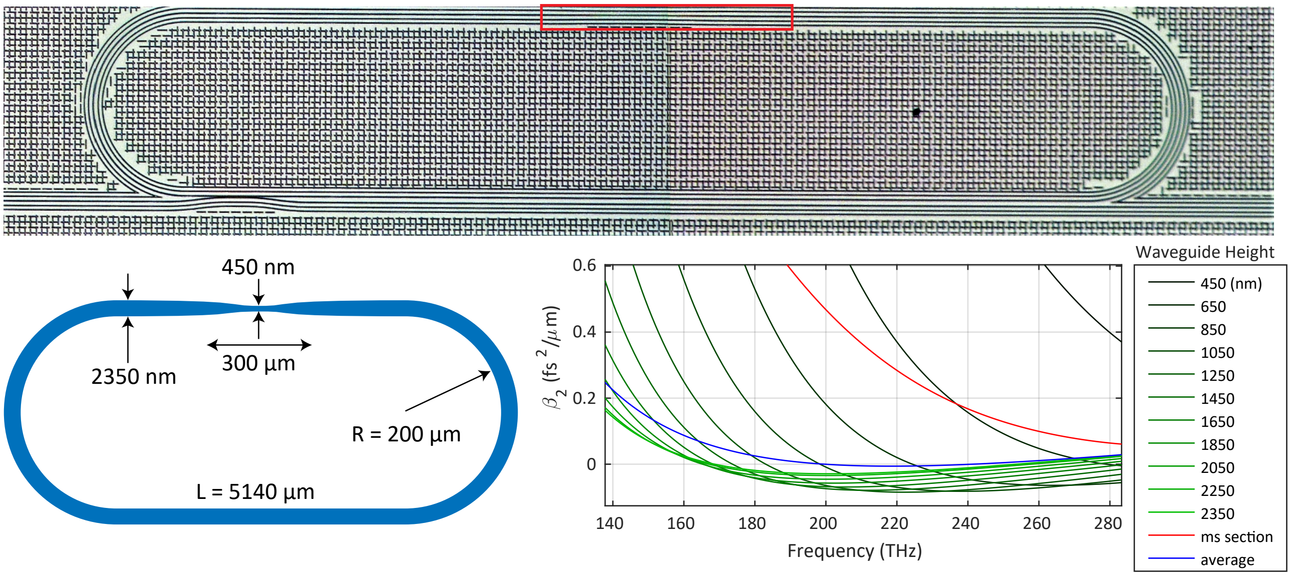

FIG. S.3. **Microresonator structure and dispersion.** Top: microscope image of Si_3N_4 microresonator, highlighting location of mode-stripping section. Left: schematic of waveguide length, width. Right: dispersion values for Si_3N_4 waveguide with a height of 710 nm and varying width, and including average dispersion value for the mode-stripping section and the whole resonator in aggregate.

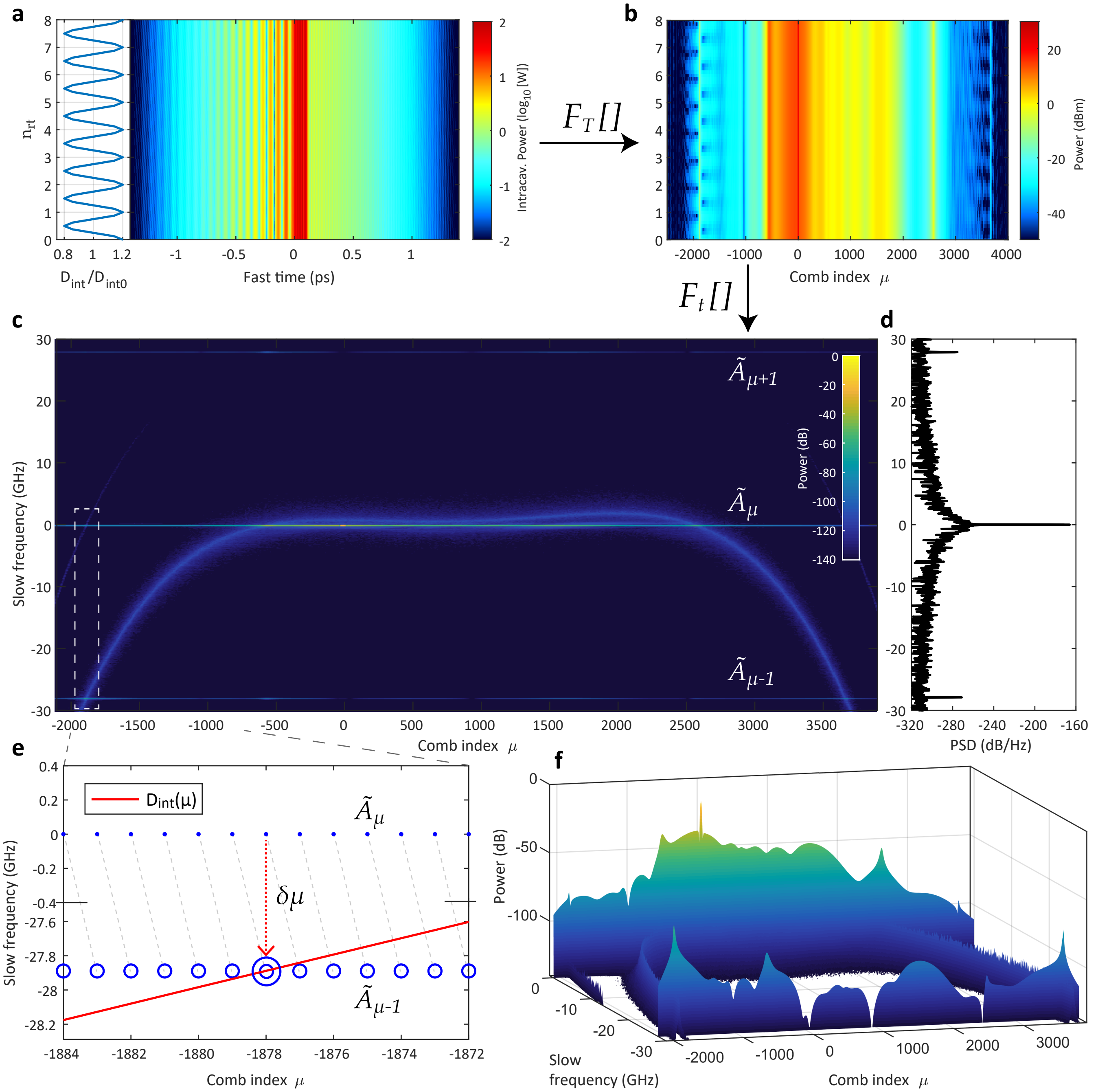

FIG. S.4. **Temporal super-resolution simulation for ZDS propagation with quasi-phase matching.** (a) Slow time vs. fast time image of ZDS propagation, with varying dispersion 'amplitude' shown on the left. (b) Slow time vs. fast frequency (comb index μ) image. (c) Slow frequency vs. fast frequency image. White box depicts section in the next panel. (e) Diagram for momentum mismatch, with quasi-phase matching marked with red
