# Supplementary material for: Zero dispersion Kerr solitons in optical microresonators
Source: Nat Commun. 2022 Aug 13;13:4764. doi: 10.1038/s41467-022-31916-x (PMC9376110; doi:10.1038/s41467-022-31916-x)
Supplement: Supplementary file 2 — Description to Additional Supplementary Information [file 41467_2022_31916_MOESM2_ESM.pdf]

Description of Additional Supplementary Files

Video for Figure 1: Dispersion Rotation

Adiabatic split-step simulation of the normalised Lugiato-Lefever Equation, with pulsed-drive input f(τ), for solitary dissipative structure held at the centre while the dispersion coefficients (d₂, d₃) traverse the full unit circle starting and ending at (1,0). The full duration takes t_{photon}=11,520. States observed in order include: switching wave, switching wave with dispersive wave, zero-dispersion soliton of order (6) to (2), soliton molecule, breathing soliton, soliton with dispersive wave, zero dispersion soliton of order (2) to (4), switching wave with dispersive wave, switching wave. (Top left) Unit circle and coordinate for (d₂, d₃), and intracavity energy. (Top right) Frequency domain. (Bottom left) Real and imaginary field contour. (Bottom right) Time domain field power.
